# New Appliances and Things Medical

**Published:** 1897-08-07

**Authors:** 


### NEW APPLIANCES AND THINGS MEDICAL.

### HEADPIECE FOR OPERATIONS.

One of the Surgeons at the National Hospital for Paralysis and Epilepsy has designed a headpiece for the operating table which deserves notice. The head-rest itself is irregularly shaped to afford the greatest support to the skull, and is firmly attached by a short length of metal tubing to a polished ball about the size of a cricket ball. This ball can be clamped in any position, at any angle, on an adjustable bar which pierces the table at the proper place. He has also had a pair of indiarubber sleeves made to wear during an operation, and finds it convenient to wash them at the same time as his hands.

